# Glucocorticosteroids as Dengue Therapeutics: Resolving Clinical Observations With a Primary Human Macrophage Model

**DOI:** 10.1093/cid/cis1048

**Published:** 2013-03-15

**Authors:** Andrew C. Sayce, Joanna L. Miller, Nicole Zitzmann

**Affiliations:** Oxford Glycobiology Institute, Department of Biochemistry, University of Oxford, United Kingdom

To the Editor—A recent trial [1] investigated the use of a glucocorticosteroid, prednisolone, as a therapy for reduction of severe dengue disease. Many pathogens induce accelerated or excessive inflammation, resulting in detrimental rather than protective effects [2], and dengue virus is a well-characterized example of this phenomenon. Several soluble mediators of the innate inflammatory response have been linked with severe pathology [3]; however, these studies are largely correlative and have failed to elucidate molecular mechanisms facilitating specific pathologies. Nevertheless, continued observation of excessive inflammation concurrent with a drop in viremia and development of severe symptoms [4, 5] has prompted several previous attempts at immunosuppressive strategies as a means of reducing severe dengue disease [6–9].

Enhancement of disease due to suppression of innate antiviral mechanisms has been a primary concern. The study by Tam et al [1] revealed neither protective effects (as discussed by Prof Barrett [10]) nor enhancement of disease. These results are surprising, because it seems likely that glucocorticosteroid treatment would prove either productive (ie, reducing excessive inflammation and resultant severe symptoms) or detrimental (ie, reducing innate mediators of viral clearance such that a greater incidence of severe disease would be noted). We have data that may explain why glucocorticosteroid treatment fails to affect the presentation of severe dengue.

We investigated dexamethasone as a dengue therapeutic in primary monocyte-derived macrophages from dengue-naive donors. From these samples, we collected cytokine and viral titer measurements at days 1, 3, and 5 and note an interesting phenomenon that we believe complements the results obtained in patients (Figure 1). We observed a significant (Mann-Whitney *U,* α < .05) decrease in viral load on day 1 of dexamethasone treatment, concomitant with decreased, but still elevated, levels of select inflammatory cytokines (RANTES, tumor necrosis factor α, interferon γ, interleukin 8, interleukin 10, interleukin 17A, granulocyte colony-stimulating factor, and monocyte chemoattractant protein 1). By day 3, suppression of cytokines was still evident, but viral titers had rebounded to the same level as in untreated samples. Viral titers for dexamethasone-treated samples did not exceed those in untreated samples on day 3 or day 5, presumably because the inflammatory response remained sufficiently intact to prevent enhanced viral load.

**Figure 1.**
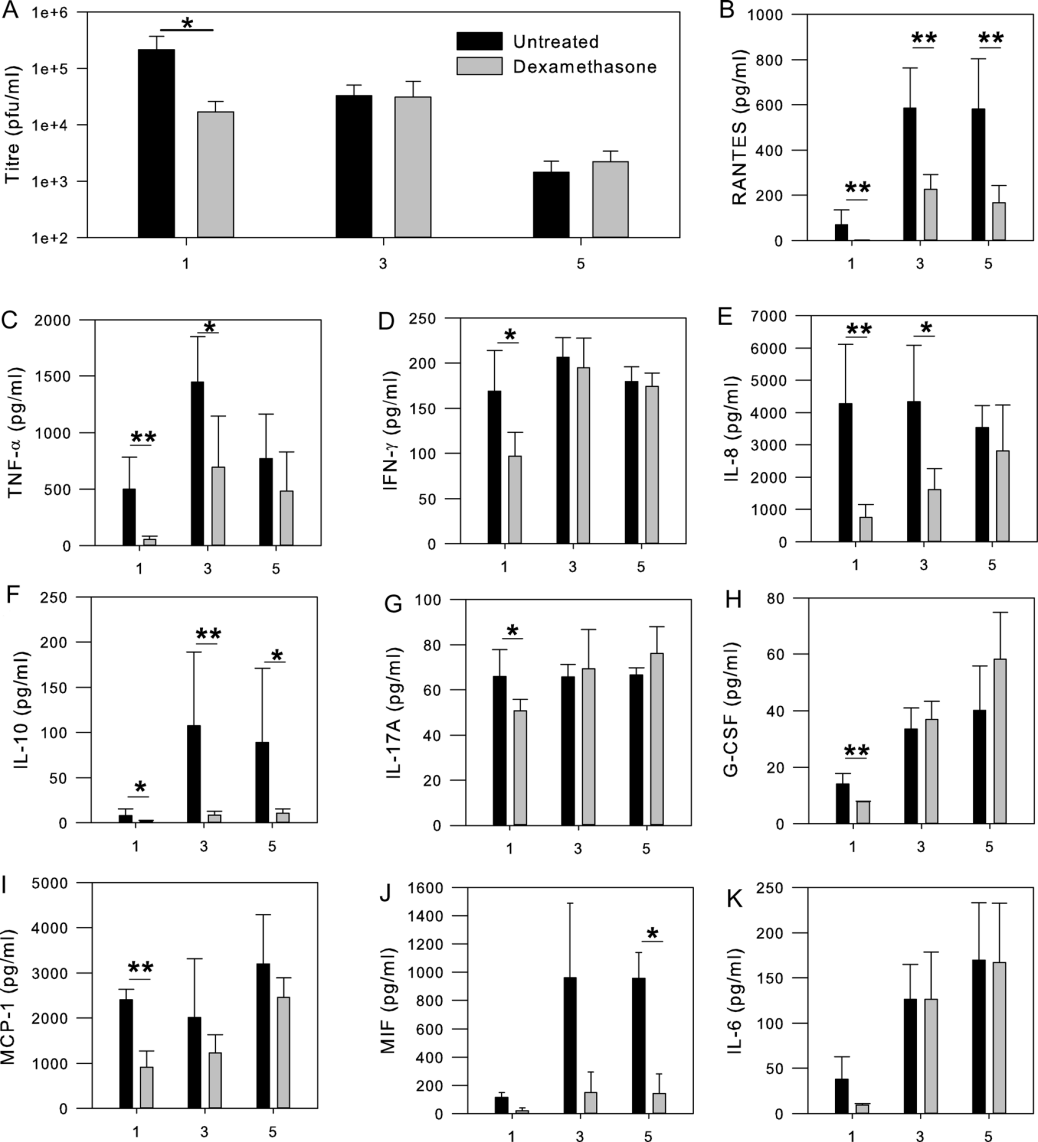
*A,* Dengue virus titer kinetics in primary monocyte-derived macrophages from 5 donors in the absence (black bars) or presence (gray bars) of dexamethasone as determined by LLC-MK_2_ plaque assay. *B–K,* Equivalent kinetic assessment of cytokines and chemokines was conducted by performing custom Bio-Plex multiplex analysis with a Luminex 200 analyzer. Error bars represent standard errors, with significance (*α < .05, **α < .01) assessed by Mann-Whitney *U* nonparametric tests, owing to failure of Levene's test for homogeneity. All x-axis values represent days after infection (days 1, 3, and 5). Abbreviations: G-CSF, granulocyte colony-stimulating factor; IFN-γ, interferon γ; IL-6, interleukin 6; IL-8, interleukin 8; IL-10, interleukin 10; IL-17A, interleukin 17A; MCP-1, monocyte chemoattractant protein 1; MIF, macrophage migration inhibitory factor; PFUs, plaque-forming units; TNF- α, tumor necrosis factor α.

This finding has implications for the use of immunosuppressive therapies against dengue and other diseases that manifest through excessive immune activation (eg, avian influenza, severe acute respiratory syndrome coronavirus). Although these drugs are capable of reducing inflammatory mediators in important targets of infection in tissues (macrophages), the systemic effects and related severe pathologies may not be affected. Alternatively, the effectiveness of treatment may depend critically on the time of drug administration. Immediate treatment following infection may be able to help direct the developing immune response to effective viral clearance while preventing severe symptoms in the subset of patients at risk of shock or hemorrhage; however, this is an unrealistic option in the clinical setting. We suggest that modest immunosuppression may be safe for use in these diseases as an adjunctive therapy in coordination with a direct-acting antiviral capable of reducing pathogen load efficiently. Future experiments investigating such a strategy may unlock the therapeutic potential of otherwise nonviable options.

## References

[1] Tam DT, Ngoc TV, Tien NT (2012). Effects of short-course oral corticosteroid therapy in early dengue infection in Vietnamese patients: a randomized, placebo-controlled trial. J Clin Infect Dis.

[2] Tisoncik JR, Korth MJ, Simmons CP, Farrar J, Martin TR, Katze MG (2012). Into the eye of the cytokine storm. Microbiol Mol Biol Rev.

[3] Rothman AL (2011). Immunity to dengue virus: a tale of original antigenic sin and tropical cytokine storms. Nat Rev Immunol.

[4] Libraty DH, Endy TP, Houng HS (2002). Differing influences of virus burden and immune activation on disease severity in secondary dengue-3 virus infections. J Infect Dis.

[5] Green S, Vaughn DW, Kalayanarooj S (1999). Early immune activation in acute dengue illness is related to development of plasma leakage and disease severity. J Infect Dis.

[6] Kularatne SA, Walathara C, Mahindawansa SI (2009). Efficacy of low dose dexamethasone in severe thrombocytopenia caused by dengue fever: a placebo controlled study. Postgrad Med J.

[7] Srichaikul T, Punyagupta S, Sorakhunpipitkul L, Udomsubpayakul U (2011). Adjunctive corticosteroid therapy in 149 grade II (non-shock) adult DHF patients: an analysis during January 2008-February 2010. J Med Assoc Thailand.

[8] Tassniyom S, Vasanawathana S, Chirawatkul A, Rojanasuphot S (1993). Failure of high-dose methylprednisolone in established dengue shock syndrome: a placebo-controlled, double-blind study. Pediatrics.

[9] Futrakul P, Poshyachinda M, Mitrakul C (1987). Hemodynamic response to high-dose methyl prednisolone and mannitol in severe dengue-shock patients unresponsive to fluid replacement. Southeast Asian J Trop Med Public Health.

[10] Barrett AD (2012). Editorial commentary: short-course oral corticosteroid therapy is not effective in early dengue infection. J Clin Infect Dis.

